# Assessment of Determinants and Causes of Preterm Births Among Postnatal Mothers: A Retrospective Study

**DOI:** 10.7759/cureus.91731

**Published:** 2025-09-06

**Authors:** Soumi Samanta, Udita Ghosh, Shruti Ghosh, Kalyani Rath, Anasuya Behera, Rojalin Dash, Monalisa Mall

**Affiliations:** 1 Obstetrics and Gynecological Nursing, Kalinga Institute of Nursing Sciences (KINS), Bhubaneswar, IND

**Keywords:** high-risk newborn, postnatal mothers, preterm birth, preterm newborn, retrospective study

## Abstract

Introduction

Preterm birth, defined as delivery before 37 weeks of pregnancy, is a serious health issue that contributes to infant illness, death, and long-term health problems. This study focused on the causes of preterm birth among mothers and the outcomes for their babies in a hospital in Bhubaneswar, India.

Methods

The researchers studied the medical records of 155 women who underwent preterm delivery. They collected data about the mothers’ age, health, pregnancy history, delivery type, baby outcomes, and mental health using a structured form. The data were analysed to find important patterns and associations.

Results

Most of the mothers were between 26 and 30 years old and came from rural areas with low income. Over half were first-time mothers, and many were overweight. The most common health problems during pregnancy were high blood pressure (43.9%), gestational diabetes (28.4%), and thyroid issues (31%). The majority of the babies were delivered by caesarean section (76.1%). Many newborns had low birth weight (49%) and required admission to the NICU (66.5%). The study found that low birth weight and longer NICU stays were significantly linked to preterm birth. Mental health issues like depression and anxiety affected nearly 59% of mothers after birth, though most (almost 90%) had family or social support.

Conclusion

The study found that preterm births are strongly linked to certain maternal health conditions, as high-risk cases can cause poor outcomes for newborns. Mothers who are overweight, have high blood pressure, diabetes, or thyroid issues are more likely to deliver their babies early. Babies born preterm are more likely to be underweight and need intensive care. Many mothers also face psychological upset and distress after their premature newborns. The study highlights the need for better antenatal and postnatal care and evidence-based practices, especially for women in rural and low-income areas. By improving access to healthcare and focusing on early identification and proper quality care during pregnancy, outcomes for both mothers and babies can be improved in India.

## Introduction

Preterm birth, defined as birth occurring before 37 completed weeks of gestation, is a significant global health issue that leads to considerable neonatal morbidity, mortality, and long-term developmental impairments [1]. An estimated 15 million premature babies are born worldwide each year, with the highest rates reaching up to 13% in India [1,2]. In 2020, the latest year with complete global estimates, approximately 13.4 million babies were born prematurely, representing 9.9% of all live births worldwide [3]. Southern Asia has the highest rate of preterm births at 13.2%, followed closely by Sub-Saharan Africa. Together, these two regions account for over 65% of all global preterm births [4]. The causes are complex and involve multiple factors, including maternal health, obstetric conditions, socioeconomic status, behavioural aspects, and environmental influences [5,6]. Both maternal age (younger than 22 or older than 35 years) and primiparity have been shown to increase the risk of preterm birth [7,8]. Short maternal height and a low pre-pregnancy body mass index (BMI) suggest inadequate nutrition and are also associated with related health concerns [6,9]. Pregnancy‑induced hypertension (PIH), gestational hypertension or preeclampsia, and prior caesarean delivery considerably raise odds [5]. Maternal infections such as bacterial vaginosis and asymptomatic bacteriuria, along with short interpregnancy intervals, multiple births, oligo- or polyhydramnios, and premature rupture of membranes, are significant obstetric risk factors [10]. Psychosocial stress, such as socioeconomic deprivation stress, racism, disasters, or maternal chrono-disruption, increasingly appears as a predictor [11]. Gestational diabetes (GD) and maternal obesity also contribute indirectly through hypertensive or metabolic mechanisms [12]. Insufficient antenatal care (<4 visits) or late booking continues to be a persistent modifiable risk [13]. Environmental exposures like ambient air pollution and heat extremes also cause low birth weight and preterm birth [14]. Preterm birth has significant short- and long-term effects on newborns, mothers, and the healthcare system. Preterm infants are at greater risk of respiratory distress syndrome, sepsis, hypothermia, hypoglycaemia, necrotising enterocolitis, intraventricular haemorrhage, and feeding difficulties [15].

In India, rural and urban studies report correlations of young maternal age (<22 years), primi-gravida, anaemia, adverse obstetric history, and suboptimal care with increased risk of preterm and low birth weight [5]. National survey statistics (NFHS‑4, 2015‑16) also correlate low antenatal care, past caesarean, and private‐facility delivery with increased preterm prevalence and higher risk of neonatal mortality in the early neonatal period [8]. Postnatally, preterm infants have an increased risk of respiratory distress syndrome, gastrointestinal, neurological, sensory, growth, and developmental morbidities [15]. This retrospective study seeks to explain maternal factors associated with preterm birth and identify the outcomes of preterm births.

## Materials and methods

This retrospective observational study was conducted to assess maternal and neonatal outcomes, with the psychological well-being of mothers during the postnatal period. The study was carried out in a tertiary care hospital located in Bhubaneswar, Odisha, India, and data were retrieved from the discharge records of postpartum women admitted between December 2023 and June 2025.

All postpartum women who delivered in the selected hospital during the study period and had complete discharge summaries were considered eligible for inclusion. Specifically, postnatal mothers with comprehensive medical and discharge records documenting both maternal and neonatal outcomes were included. In contrast, those with incomplete or missing discharge summaries or records lacking essential information were excluded.

The study examined variables across three domains. Maternal factors included age, parity, education, socioeconomic status, antenatal history, mode of delivery, and maternal complications. Neonatal outcomes were assessed in terms of birth weight, neonatal complications, and discharge outcomes. Psychological well-being was evaluated based on documented indicators of stress, anxiety, or depression recorded in the discharge summaries.

Data were extracted retrospectively using a structured 29-item questionnaire developed specifically for this study. The tool was designed to systematically capture demographic, obstetric, clinical, neonatal, and psychosocial information, thereby ensuring a comprehensive assessment of both medical and emotional aspects of postnatal health. The study size was determined by the number of complete discharge summaries available during the specified period, and no prior sample size calculation was undertaken.

Data entry was performed using Microsoft Excel, and statistical analyses were carried out using IBM SPSS Statistics for Windows, Version 25 (Released 2017; IBM Corp., Armonk, New York, United States). Descriptive statistics were used to summarise the data, with means and standard deviations reported for continuous variables, and frequencies with percentages for categorical variables. Associations between categorical variables were tested using the chi-square test and Fisher’s exact test, and a p-value of less than 0.05 was considered statistically significant.

## Results

Socio-demographic profile

Most participants were aged 26-30 years (33.5%). The majority had a high school certificate (37.4%), were unemployed (44.5%), and belonged to the upper-lower (33.5%) or lower-middle class (32.9%). Geographically, most lived in rural areas (63.9%).

Maternal outcome

Most deliveries occurred at 33-37 weeks of gestation (60.6%). Regarding BMI, 41.3% were overweight. The most common complications were PIH (43.9%) and thyroid disorders (31%). Prenatal care was received by 63.2%. A majority were delivered by caesarean section (76.1%), and 43.9% stayed in the hospital for 3-7 days.

Fetal outcome

Nearly half of the babies were low birth weight (49%), and 66.5% required NICU admission. In terms of maternal psychological health, 58.7% experienced depression and anxiety, though 89.7% received psychological support.

Association between preterm birth and maternal-neonatal outcomes

Based on the Pearson chi-square and Fisher’s exact test results, most of the examined variables did not show statistically significant associations with preterm status. Specifically, there were no significant relationships between preterm delivery and mode of delivery (p = 0.627), delivery complications (p = 0.552), ICU admission (p = 0.659), postpartum complications (p = 0.971), maternal psychological outcomes such as depression and anxiety (p = 0.287), or need for psychological support (p = 0.873). Similarly, the duration of maternal hospital stay (p = 0.056) and NICU admission (p = 0.057) approached significance but did not meet the conventional threshold (p < 0.05). However, two variables showed statistically significant associations with preterm birth: the baby’s weight (p = 0.032) and the duration of NICU stay (p = 0.019). This suggests that while most maternal outcomes were not significantly affected, neonatal outcomes, specifically lower birth weight and longer NICU stays, were significantly associated with preterm status.

**Table 1:** Association Between Preterm Birth and Maternal–Neonatal Outcomes Assessed Using Pearson Chi-Square and Fisher’s Exact Tests This table presents the association between preterm birth and selected maternal and neonatal outcomes. Analyses were performed using Pearson’s chi-square test, with Fisher’s exact test applied when expected cell counts were small. Test Value: Chi-Square or Fisher’s statistic; df: Degrees of freedom; P-value: Probability of observing the results under the null hypothesis of no association. Statistical significance: A p-value < 0.05 was considered statistically significant. Significant associations were observed for the birth weight of the baby (p = 0.032)* and the duration of NICU stay (p = 0.019)*, indicating that preterm birth was significantly related to these outcomes. All other variables did not show statistically significant associations. Results marked with an asterisk denote significance at the 0.05 level.

For preterm	Test Value	df	P-value
Mode of delivery	0.933	2	0.627
Complications during delivery	0.355	1	0.552
ICU stay	0.195	1	0.659
Postpartum complication	0.237	3	0.971
Duration of hospital stay mother	5.757	2	0.056
Weight of the baby	6.874	2	.032*
Admitted to the NICU	3.621	1	0.057
Stay in the NICU	9.990	3	.019^*^
Depression and anxiety	1.133	1	0.287
Psychological support	0.026	1	0.873

## Discussion

Participants were aged between 26 and 30 years, had a high school education, and lived in rural towns characterised by low socioeconomic status, which could have affected access to good-quality maternal healthcare services. Most women were unemployed, which could potentially restrict their autonomy in healthcare decision-making. Obstetric findings indicated a high frequency of primigravida and primipara mothers, with a large percentage giving birth preterm (33 to 37 weeks), which indicates the susceptibility of first-time mothers to negative outcomes. The high proportion of caesarean sections (76.1%) to vaginal deliveries indicates an increasing trend towards surgical delivery, possibly due to medical complications or institutional policies. Moreover, a high percentage of the mothers were overweight and had conditions like PIH, gestational diabetes mellitus (GDM), and thyroid disease, all of which are established risk factors for preterm labour and neonatal morbidity.

Neonatal outcomes were also seen to be greatly influenced by birth weight and NICU admission, with low birth weight and long NICU stays being prevalent. There were evident mental health issues, with more than half of the mothers describing depression and anxiety, although the majority had psychological support. These results indicate the urgent need for gestational and postpartum comprehensive care, particularly for those at risk, in conjunction with mental health advice and prompt detection of complications. The statistically significant relationships between preterm birth and neonatal outcomes highlight the value of enhancing maternal and neonatal health in comparable groups.

One Swedish study showed similar consequences where 226 children were born before 27 weeks and found that 37% had developmental coordination disorder (DCD) at age 6½. Key risk factors included older maternal age, pre-eclampsia, mothers born outside the Nordic region, lower gestational age, retinopathy of prematurity, postnatal steroid use, and mechanical ventilation. The findings show that extremely preterm children face a very high risk of DCD due to common medical and pregnancy-related complications in this group [15].

Our results align with earlier studies showing that preterm birth is strongly linked to maternal medical conditions and obstetric risk factors. Both studies demonstrated that complications such as hypertension, diabetes, and a history of preterm birth contribute significantly to early deliveries, while neonatal outcomes are largely determined by low birth weight and NICU admission. Importantly, both findings emphasise that many preterm deliveries stem from potentially preventable causes, highlighting the need for improved maternal care, timely interventions, and closer monitoring to reduce avoidable adverse outcomes [16].

The two studies offer complementary information on the determinants of preterm birth and its consequences. The large Italian cohort from University Hospital "G. Martino" reported well-known biomedical predictors like abnormal maternal BMI, hypertensive disorders, diabetes, uterine malformations, and short cervical length, confirming the importance of clinical monitoring. In contrast, the small Indian rural cohort of mothers placed key emphasis on the burden of sociodemographic limitations, excessive caesarean delivery rates, and the psychosocial morbidity of anxiety and depression, highlighting how contextual risk factors amplify biological vulnerabilities. Both studies, though differing in design and population, come together on the premise that preterm birth is a result of a cluster of modifiable risk factors. This convergence underscores the requirement for combined strategies that pair medical intervention with social and psychological care in order to enhance maternal and neonatal health [17].

Preterm birth is closely linked to negative outcomes for both mothers and infants. Infants born preterm face increased risks of mortality, respiratory distress, intraventricular haemorrhage, and long-term neurodevelopmental issues. Generally, these outcomes improve with greater gestational age and higher birth weight. For mothers, complications can arise from conditions such as hypertension, GD, and infections, leading to difficulties during delivery and increased postpartum health problems. Additionally, mothers of preterm infants often experience heightened psychological distress, particularly in the form of anxiety and depression, highlighting the dual challenges posed by preterm birth [18].

This study has several limitations. First, it was retrospective, relying solely on hospital discharge summaries, which may contain missing or incomplete information, especially concerning lifestyle and psychological aspects. Second, the sample size was relatively small (155 discharge summaries), which may limit the ability to detect smaller differences. Third, only mothers with complete hospital records were included, which could lead to selection bias and exclude home deliveries. Fourth, the study assessed outcomes only up to discharge, without conducting long-term follow-up for mothers or babies. Finally, depression and anxiety data were based on limited notes or self-reporting, without standardised mental health assessments.

Future studies should be prospective and include multiple hospitals, including rural and primary health centres, to make findings more generalizable. Data collection should encompass nutrition, family support, and lifestyle factors to provide a more comprehensive picture. Standardised tools should be used to assess mental health. Antenatal care programs must focus on high-risk mothers, especially those with conditions like PIH or GDM. Community outreach can improve healthcare access for rural and low-income women.

## Conclusions

This retrospective study highlights key maternal and neonatal factors associated with preterm births among postnatal mothers in a selected hospital in Bhubaneswar, India. A significant proportion of mothers were young, primigravida, overweight, and from socioeconomically disadvantaged rural backgrounds, all of which contributed to increased risk of preterm delivery. Medical complications such as PIH, GD, and thyroid disorders were prevalent. Caesarean delivery was common, reflecting either clinical necessity or institutional preference. Neonatal outcomes, particularly low birth weight and prolonged NICU stays, were significantly associated with preterm births. Furthermore, maternal psychological distress was prominent, underscoring the need for integrated mental health support. The findings suggest appropriate systematic antenatal care, early risk identification, and targeted interventions addressing both medical and psychosocial factors. Enhancing healthcare accessibility and quality, evidence-based care, especially for rural and low-income populations, is essential to reduce the burden of preterm births and improve maternal-infant outcomes.

## Appendices

Questionnaire

Section 1: Sociodemographic Information

1. Age of the mother: (a) < 20 (b) 21-25 (c) 26-30 (d) 31-35 (e) > 35 2. Marital Status: (a) Single (b) Married (c) Divorced/Widowed 3. Educational Level: (a) professional or honours (b) graduate (c) intermediate or diploma (d) High School Certificate (e)Middle School certificate (f) Primary school certificate (g) Illiterate 4. Occupation: (a) Legislators, senior officials and managers (b) Professionals (c) Technicians and associate professionals (d) Clerks (e) Skilled workers and shop and market sales workers (f) Skilled agricultural and fishery workers (g) Craft and related trade workers (h) Plant and machine operators and assemblers (i) Elementary occupation (j) Unemployed 5. Socioeconomic Status:

(a) Upper (b) Upper middle (c) Lower Middle (d) Upper Lower (e) Lower 6. Place of Residence: (a) Urban (b) Rural (c) Slum

*Section 2: Obstetric History and Pregnancy-Related Information* 7. Gravida (Total number of pregnancies): (a) 1 (b) 2-3 (c) 4 or more 8. Parity (Number of live births): (a) 1 (b) 2-3 (c) 4 or more 9. Gestational Age at Birth: (a) < 28 weeks (b) 28-32 weeks (c) 33-37 weeks 10. What was your BMI before delivery? a) Underweight: BMI less than 18.5 b) Normal weight: BMI between 18.5 and 24.9 c) Overweight: BMI between 25 and 29.9 d) Obesity (Class 1): BMI between 30 and 34.9 e) Obesity (Class 2): BMI between 35 and 39.9 f) Severe Obesity (Class 3): BMI 40 and above 11.Complications during pregnancy: a) Hypertension b) Gestational diabetes c) Placenta previa d) Pre-eclampsia e) No complications f) others(specify) 12.Were you diagnosed with any infections during pregnancy? (Please select all that apply) a) Urinary tract infection (UTI) b) Sexually transmitted infections (STIs) c) Vaginal infections d) Respiratory infections (e.g., pneumonia) e) Other (please specify) ___________ f) None 13. Was prenatal care adequate and regular? (a) Yes (b) No (c) Not sure 14. Presence of any medical conditions in the mother (pre-existing or during pregnancy):

(a) Hypertension (b) Diabetes (c) Asthma (d) Thyroid disorder (e) other (specify) 15. Was there any history of previous preterm births? (a) Yes (b) No 16. Were you carrying multiple pregnancies (e.g., twins, triplets)? (a)Yes (b)No 17. Did you experience premature rupture of membranes (PPROM) during pregnancy? (a)Yes (b)No

*Section 3: Delivery-Related Information* 18. Mode of Delivery: (a) Vaginal delivery (b) Caesarean section (c) Assisted delivery (Forceps/Vacuum) 19. Any complications during delivery? (a) Yes (b) No If yes, specify: _______ 20. Was the mother transferred to the Intensive Care Unit (ICU)? (a) Yes (b) No If yes, reasons for transfer: _______

*Section 4: Maternal Health Outcomes* 21. Postpartum complications: (a) Haemorrhage (b) Infection (c) Wound complications (e) None 22. Duration of hospital stay for the mother: (a) < 3 days (b) 3-7 days (c) 7+ days

*Section 5: Neonatal Outcomes* 23. Birth Weight of the Newborn: (a) Low birth weight (< 2.5 kg) (b) Normal birth weight (2.5 kg - 4 kg) (c) High birth weight (> 4 kg) 24. Was the neonate admitted to the Neonatal Intensive Care Unit (NICU)?

(a) Yes (b) No 25. Duration of NICU stay: (a) < 1 week (b) 1-3 weeks (c) > 3 weeks

Section 6: Psychological Impact 26. Did the mother experience postpartum depression or anxiety? (a) Yes (b) No 27. Did the mother receive psychological support or counselling? (a) Yes (b) No - As it was a self-structured tool, two questions were excluded before data analysis. They were: 1) Follow up care a) Yes b) No 2) Any additional factors that could have contributed the preterm birth a) Yes b) No
